# Specification of germ layer identity in the chick gastrula

**DOI:** 10.1186/1471-213X-7-91

**Published:** 2007-07-30

**Authors:** Susan C Chapman, Kiyoshi Matsumoto, Qin Cai, Gary C Schoenwolf

**Affiliations:** 1 University of Utah School of Medicine, Department of Neurobiology and Anatomy, and Children's Health Research Center, Room 2R066 SOM, 30 North 1900 East, Salt Lake City, Utah, 84132-2101, USA; 2 Clemson University, Biological Sciences, 340 Long Hall, Clemson, SC, 29634, USA; 3 Development Research Center, Pharmaceutical Research Division, Takeda Pharmaceutical Company Limited, 17-85, Jusohonmachi 2-chome, Yodogawaku, Osaka 532-8686, Japan

## Abstract

**Background:**

Chick definitive endoderm is an important source of signals that pattern the early embryo forming a central structure around which the body plan is constructed. Although the origin of definitive endoderm has been mapped in the chick, arising principally from rostral streak at elongating streak stages, it is not known when this layer first becomes fully committed to its germ layer fate, an important issue to resolve in light of its critical role in subsequent patterning of the early embryo.

**Results:**

Through gene expression screening of chick gastrula, we identified molecular markers of definitive endoderm restricted to rostral (*Sox17*) and caudal (*Gata5/6*) regions, suggesting that at least two subpopulations of definitive endodermal cells exist during ingression. We show (1) that presumptive mesoderm cells migrate to the middle layer and remain mesenchymal when transplanted to rostral primitive streak, and prospective endoderm cells enter the lower layer and become epithelial when transplanted to caudal primitive streak; and (2) that presumptive endoderm cells and mesoderm cells lose normal gene expression (*Sox17 *and *Wnt8c*, respectively) when transplanted outside of their normal position of origin. Moreover, when rostral or caudal primitive streak segments are transplanted into rostral blastoderm isolates (RBIs), both types of transplants express *Sox17 *4–6 hours later–consistent with their new position, regardless of their presumptive germ layer origin–and prospective mesoderm transplants, which normally express *Wnt8c*, turn off expression, suggesting that signals within the rostral blastoderm induce endoderm gene expression, and repress mesoderm gene expression, during gastrulation.

**Conclusion:**

Our results demonstrate that germ layer identity is fixed at the time populations of endoderm and mesoderm cells ingress through the primitive streak, whereas their gene expression patterns remain labile. In addition, our results show that inductive and repressive signals are present, and that these signals regulate gene expression of both ingressed endoderm and mesoderm cells. Thus, gastrula cells display elements of both pre-patterning and plasticity, with endoderm the first germ layer becoming committed to its fate during early gastrulation stages.

## Background

The endoderm is a source of signals that pattern anterior structures [1,2], facial skeleton [3], heart [4,5], left-right heart asymmetry [6] and inner ear development [7]. Formation of endoderm has been studied in a number of animal models, for example, in *Xenopus*, maternally derived VegT acts via Nodal signaling upstream of Mix, Gata and Xsox17 in specification of definitive endoderm. Later markers of endoderm include *Cerberus*, *endodermin*, and *Xhex *[8,9]. Similarly in zebrafish, Nodal signaling involves Gata5 and Mixer in activation of *Sox17 *expression via the zebrafish-specific *Casanova *gene related to *Sox17 *[10,11]. A recent microarray study in *Xenopus *has revealed some 300 endoderm-expressed genes, with identification of a number of novel Nodal, Mixer and Sox17 proteins [12]. However, with less than 10% of the endoderm transcriptome being regulated as predicted, the linear model of endoderm development is under renewed scrutiny.

Development of the chick embryo between unincubated prestreak stages (stage 1) and definitive streak at stage 4 is very dynamic. Primitive endoderm, consisting of primary hypoblast (endophyll) delaminating from the epiblast through polyingression toward the subgerminal cavity, together with the rostrally migrating endoblast (secondary hypoblast/sickle endoblast) originating from Koller's Sickle (KS), forms a continuous sheet of primitive endoderm that underlies the epiblast at EGK stage XIV [13], prior to formation of the primitive streak at stage 2. At stage 1, the most posterior embryonic tissues consist of three populations: KS, the posterior marginal zone (PMZ) and the caudal germ wall (CGW). Examination of sectioned embryos shows that each of these three populations consist of multiple layers of cells: superficial (epiblast), middle and deep cells in KS, the PMZ and the CGW. Middle cells are sandwiched between the epiblast layer and the deep cells, which are in direct contact with the yolk. With formation of the primitive streak, definitive endoderm begins to ingress through the rostral streak [14-17], displacing hypoblast, which is fated to become extraembryonic tissue. Replacement of the lower layer is essentially completed by the time the streak has reached maximal extension at stage 4 [1].

In chick, little is known about the molecular signaling pathways involved in specifying definitive endoderm. To begin addressing this question we have used 1) in situ hybridization (ISH) of potential endodermal markers to screen the expression patterns of several chick orthologues, 2) heterotopic quail to chick streak transplants and 3) quail primitive streak transplants into chick rostral and caudal blastoderm isolates.

## Results and discussion

### In situ hybridization analysis of putative endoderm marker genes

**Figure 1:**
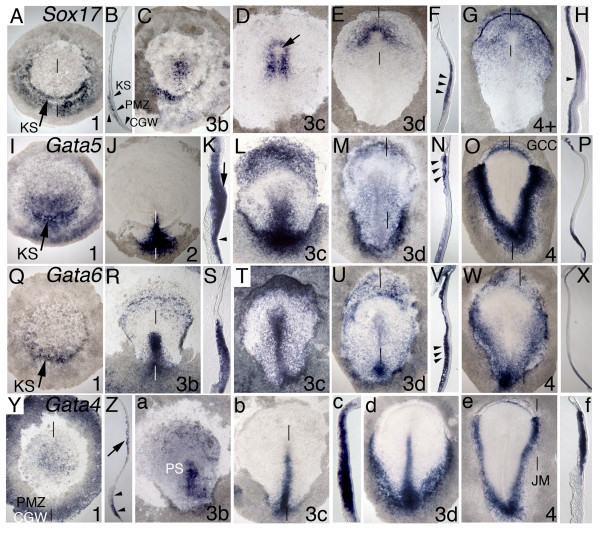
**Molecular markers reveal subpopulations of definitive endoderm**. Whole mount embryos and sections of *Sox17 *(A-H), *Gata5 *(I-P) and *Gata6 *(Q-X) and *Gata4 *(Y, Z, a-f), analyzed by in situ hybridization (ISH). Stages in bottom right hand corner, anterior to the top, 50 μm thick sagittal sections, lines on whole mount image indicates level of section. *Sox17*: (A-H) *Sox17 *in a prestreak blastoderm stage embryo has dynamic expression within Koller's sickle (KS, arrow) with the rostrally extending sickle horns clearly defined by its expression (A). (B) The posterior marginal zone (PMZ, arrowheads) has transcripts in the middle layer, whereas the caudal germ wall (CGW, arrowheads) is negative through all layers. (C) Primitive streak has formed, extending rostrally with *Sox17 *expressed in a subset of rostral streak cells and in Koller's sickle. (D) Koller's sickle expression down regulates at stage 3c, leaving only the rostral streak positive for transcripts. Note that the central area of anterior tip cells is negative for *Sox17 *transcripts (arrow). (E) By stage 3d, *Sox17*-positive endoderm is detected in the early rostral ingressing definitive endoderm (de), anterior and lateral to Hensen's node. (F) In section (arrowheads) definitive endoderm in the lower layer. (G) Maximal streak extension is reached at stage 4+, with definitive endoderm having displaced the hypoblast to the extraembryonic region. Only rostral definitive endoderm now expresses *Sox17*, with all the definitive endoderm having exited the streak. Axial mesoderm has begun to ingress at stage 4+ (arrowhead in H). *Gata5*: (I-P) *Gata5 *expression is strongest in Koller's sickle (KS), with diffuse expression in the posterior hypoblast, PMZ and CGW (I). (J, K) At streak formation (stage 2) restricted expression is detected the caudal two-thirds of the primitive streak, KS (arrow), epiblast PMZ (arrowhead), but not in the CGW (J). (L-N) By stage 3c/d, rostrally displaced hypoblast is positive for transcripts in an initially wide rostral band (arrowheads in section M). For all Gata genes the germinal crescent cells (GCC) are positive (O, W, e), with *Gata5 *having the best-defined crescent (O, P). Note the broad streak expression (L), spreading laterally as mesoderm and endoderm migrate away (M). (O, P) By stage 4, the central area pellucida is negative for transcripts, with the germ cell crescent (GCC) well defined and junctional mesoderm and endoderm at the lateral edge of the area pellucida strongly positive for transcripts, forming a sharp line at the level of the heart field. *Gata6*: (Q-X) For *Gata6*, restricted expression in KS and the lateral sickle horns are well defined (Q). From stage 3b (R), rostrally situated hypoblast has mosaic expression as do all layers of the streak (S). By stage 3c/d (R-V), caudally ingressing endoderm and mesoderm have expression (arrowheads in V). Expression of *Gata5 *and *Gata6 *diverges by stage 4, with *Gata6 *(W, X) marking the a more caudal definitive endoderm subpopulation in addition to a less well-defined junctional population, whereas *Gata5 *(O) is strongly defined in the lateral endoderm and mesoderm (junctional) population and the center of the embryo is negative for transcripts (compare O, W). *Gata4*: (Y) Central hypoblast of prestreak embryos expresses *Gata4 *(see lower layer cells arrowed in Z), with weaker PMZ staining than CGW around the border of the blastoderm (arrowheads). (a) The primitive streak (PS) expresses strongly at stage 3b, except for the anterior tip, as well as diffuse transcripts throughout the area pellucida, becoming restricted to the middle layer of the streak by stage 3c and excluded from the (b, c). (d) The lateral (junctional) mesoderm and primitive streak, exclusive of anterior tip, has strong *Gata4 *expression by stage 3d. (e, f) By stage 4, with maximal streak extension, expression is lost in the streak, with a fine line of germ cell crescent having expression, and a narrow band of positive junctional mesoderm (JM) cells, similar to *Gata5*, extending caudally from the heart field. Endoderm cells are negative for transcripts (c, f).

A number of genes have been implicated in endoderm specification in *vertebrates *(Table 1) [18]. We examined the chick orthologues of a number of potential definitive endoderm markers using in situ hybridization (ISH) to determine their expression patterns (i.e., *Gata4, 5, 6, Sox17, Foxa2/Hnf3beta, Hnf4alpha, Mix, Edd, MafA*). Of particular interest were *Sox17 *and *Gata5 *and *6 *(Figure 1), all of which have expression in the definitive endoderm, but have not previously been analyzed at gastrulation stages.

**Table 1:** Endoderm markers and chick orthologues

Name	Synonym	Domain		Genbank	EST or Ensembl number
Gata4		Zinc finger protein	G	XM_420041	
Gata5	Faust	Zinc finger protein	G	U11888	
Gata6		Zinc finger protein	G	U11889	
Foxa1	Hnf3a Tcf3a	Forkhead-domain factor	X	NM_204088	No chick orthologue
Foxa2	Hnf3b Tcf3b?	Forkhead-domain factor	G	NM_204770	
Hnf1b	vHNF1 Tcf2	Homeobox	D H X	AF244140 X71348 XLXLFB3	ENSGALG00000005504
Hnf4a		Zinc finger domain	G	BG711675	pg11n.pk0008.m3
Hnf6	Onecut1	Homeobox	H	NM_004498	ENSGALG00000004551
Casanova	Cas Sox7?	Sox	D	AAK14780	
XSox17a1/2		Sox	G	BM439840	pgr1n.pk001.g24
XSox17b		Sox	X	AAT71997	
CMIX	Mix.1	Paired-like homeodomain factor	X G	P21711 NM204990	
Mix.2	tMix	Paired-like homeodomain factor	X X	Q91685 AAC60020	No chick orthologue
Mixer	Mix.3	Paired-like homeodomain factor	X	AF068263	No chick orthologue
Milk	Bix2	Paired-like homeodomain factor	X X	AF005999 AF079560	No chick orthologue
Bix1	Mix.4	Paired-like homeodomain factor	X	AF079559	No chick orthologue
Bix3		Paired-like homeodomain factor	X	AF079561	No chick orthologue
Bix4	tBix	Paired-like homeodomain factor	X	AF079562	No chick orthologue
Cerberus		TGFbeta signal antagonist	G	AF139721	
Endodermin	Edd		X G	L63543 NW_100702	Similar to alpha-2-macroglobulin ChEST251e24 ChEST729e9 ChEST400g4 no orthologue
Eomesodermin	Eomes	T-box factor	M	O54839	ENSGAGL00000011424
Edd	Ubiquitin ligase	HECT domain	G G G	BU399139 BU412297 BU210891	ChEST492h4 ChEST167b13 ChEST39l14
Hex	Prh Xhex	Homeobox	G	Q05502	
Lim1	Lhx1 Xlim1	Cysteine rich motif -LIM domain	X G	X63889 L35569	ENSGALG00000005409
mafA		Basic-leucine zipper (bZIP) transcription factor	G	NM_205025	
Pax6		Paired box, and homeodomain	G	NM_205066	
Tbx6L	VegT, TbxL	T-box factor	G X	Gi62554175 NM_203527	ENSGALG00000006374
Xlhbox8	Ipf1 Pdx-1 Idx-1 Stf-1 Iuf-1	Homeobox	X G	X16849 XM_425635	

### *Sox17 *gene expression

Definitive endoderm is marked by the expression of the Sry-related HMG box gene, *Sox17*, in *Xenopus*, mouse and zebrafish. We have identified the chicken orthologue of *Sox17 *(University of Delaware EST, pgr1n.pk001.g24) and analyzed its expression. Consistent with other animal models, we find that chick *Sox17 *is expressed in definitive endoderm ingressing through the rostral streak. Expression is also detected in earlier populations of posterior cells before streak formation, and in a small number of early hypoblast cells, middle layer KS cells and PMZ cells, but neither is detected in the superficial/epiblast layer of KS or of the PMZ. None of the CGW layers express *Sox17 *(Figure 1A, B). Furthermore, the middle layer cells contacting the superficial layer (i.e., middle KS and PMZ cells) express *Sox17*, whereas those contacting the yolk do not.

As the streak forms, *Sox17*-positive cells can be detected at its rostral end (Figure 1C, D). With formation of the primitive streak, *Sox17 *expression is down regulated in KS and the PMZ. By stage 3d (Figure 1E, F), only definitive endoderm expresses *Sox17 *as it ingresses and moves cranially to underlie the newly specified neural plate by stage 4+ (Figure 1G, H). Further ISH analysis [see Additional file 1], demonstrates that *Sox17 *is a transient marker of definitive endoderm, quickly becoming down regulated between stages 4 and 5 to a small number of cells in the prechordal plate, a derivative of Hensen's node, that ingresses as the neural plate undergoes shaping.

Surprisingly, *Sox17 *positive definitive endoderm cells are confined to the rostral blastoderm, whereas more caudal endoderm is negative for expression (Figure 1E, G). This could be due to definitive endoderm arising from two distinct sources, or all endoderm arising from one source, with *Sox17 *subsequently turning on in only the rostral population. Labeling of the rostral streak with fluorescent dye markers reveals that the epiblast derived definitive endoderm ingressing through the anterior streak displaces the hypoblast and underlies the entire area pellucida [14]. Thus, rostral and caudal definitive endoderm seem to have differing molecular identities on exiting the streak, suggesting that the embryo may exhibit differences in its rostral and caudal patterning capability at early stages.

The dynamic expression pattern of *Sox17 *in chick is similar to that of mouse *Sox17 *expression [19], where definitive endoderm at the anterior end of the primitive streak expresses *Sox17 *de novo. Loss-of-function mutation of Sox17 in mice reveals that redundant patterning of definitive endoderm, perhaps by other F group members, Sox7 and Sox18, allows for correct formation and patterning of anterior definitive endoderm, but later survival of foregut endoderm and differentiation of mid- and hindgut endoderm is adversely affected [19]. These results suggest that the molecular identity of rostral and caudal definitive endoderm are inherently different from the onset of their formation. In *Xenopus*, *Sox17 *expression is induced by VegT and maintained by Nodal signals in vegetal cells, resulting in endoderm identity, whereas Nodal induced by VegT in marginal zone cells lacking *Sox17 *expression become mesoderm [20]. Furthermore, Sox17 and beta-catenin can interact directly to regulate downstream endodermal gene expression of *Foxa1 *(*Hnf3alpha*) and *Foxa2 *(*Hnf3beta*) [21]. Thus, Sox proteins act as Wnt/b-catenin effectors in a similar manner to the Tcf/Lef family of HMG box transcription factors within the WNT/beta-catenin signaling pathway. Other members of the Sox family may be acting similarly as both activators and repressors of downstream gene expression.

In zebrafish another Sox gene has been identified, *Casanova *(*Cas*/*Sox32*) belonging to the F subgroup together with *Sox17 *and *Sox18*, that plays a critical role in endoderm induction. However, *Cas *has not been identified in other vertebrates. Futaki and co-workers have shown that Sox7 acts upstream of Gata factors and is the functional equivalent of zebrafish *Cas *[22]. However, Sox7 is insufficient by itself to induce Gata factors. In zebrafish, spg (Pou2/Oct4) acts synergistically with Cas and is essential for endoderm formation [23,24]. Consequently, it is likely that in both mammals and chick a SOX-POU interaction is required to induce Gata factors, but as no orthologues of *Cas*, *Sox7 *or *Pou2 *have yet been identified in the chicken genome, this is unconfirmed.

### *Gata *factors as markers of endoderm in chick

*Gata4, 5 *and *6 *(gift of Todd Evans) [25] are zinc finger transcriptional activators known to be important in endoderm specification [18]. The expression patterns of chick *Gata *orthologues at blastula/gastrula stages have not been reported previously. In both zebrafish and *Xenopus*, Nodal activates expression of *Gata5 *(Figure 1I–P), which in turn is able to initiate expression of *Sox17 *[18]. In the chick, *Gata5 *is dynamically expressed in a subset of endoderm and mesoderm tissues. At stage 1, in the posterior half of the embryo, the upper layer epiblast, KS, posterior marginal zone (PMZ) and caudal germ wall (CGW) have expression (Figure 1I). Mosaic posterior hypoblast expression and KS expression is detected, with the deepest layer of KS, the PMZ and the CGW negative for expression. Both prospective endoderm and mesoderm have expression as the streak forms at stage 2 (Figure 1J, K), with the epiblast expression now downregulated in the CGW. By stage 3c/d, hypoblast, displaced rostrally to the germ cell crescent, strongly expresses *Gata5*, as does the primitive streak endoderm and mesoderm exiting the streak (Figure 1L–N). By stage 4, significant amounts of endoderm and mesoderm have exited the streak, with *Gata5*-positive tissue migrating, forming lateral (junctional) mesoderm and underlying endoderm at the lateral margins of the area pellucida, and extending caudally to the posterior end of the primitive streak (Figure 1O, P). The rostral cells of the lateral mesoderm are fated to become heart mesoderm. The hypoblast expressing *Gata5 *forms a defined rostral germ cell crescent (Figure 1O, P).

At prestreak stages, *Gata6 *expression is restricted to middle layer KS and a small number of endoderm cells close to the sickle (Figure 1Q). No expression is detected in the epiblast. Between stages 2 and 4, the expression pattern of *Gata6 *is similar to that of *Gata5*, with less intense expression in the lateral mesoderm and caudal endoderm population, and with a less compact subpopulation of hypoblast expression in the rostral crescent (Figure 1R–X).

In F9 cells, Gata4 and Gata6 act redundantly to induce early endoderm lineages, inducing downstream *hepatocyte nuclear factors *(*Hnf1b *and *3b*) and *Sox17 *[22]. In chick, *Gata4 *is expressed in the area opaca germ wall and in primitive endoderm in unincubated chick embryos (Figure 1Y, Z). By stage 3b, the primitive streak has strong expression, with more diffuse epiblast expression (Figure 1a) that quickly diminishes in stage 3c embryos in all but the primitive streak (Figure 1b, c). The lateral mesoderm and primitive streak have expression at stage 3d (Figure 1d), and by stage 4 the streak is negative for transcripts, leaving only a thin ring of peripheral expression in the rostral endoderm of the germ cell crescent and most a narrow band of lateral (junctional) mesoderm (Figure 1e, f). Careful examination of sections indicates that all endoderm is negative for *Gata4 *expression by stage 4.

In summary, *Gata *factors are important transcription factors in endoderm identity in early embryos. In chick, they are expressed in distinct spatially restricted patterns at unincubated stages. Later expression continues in the germ cell crescent and peripheral endoderm and mesoderm, but not in the more central definitive endoderm. All three Gata factors are reported to be inducers of the definitive endoderm marker *Sox17*, with *Gata5 *and *6 *expression in unincubated chick embryos corresponding most closely to the expression of *Sox17 *(Figure 1A, I, Q). By stage3/4, *Gata6 *is expressed within the ingressing definitive endoderm in a pattern that is complementary to that of *Sox17 *(compare Figure 1E, U and 1G, W). Thus, two definitive endoderm subpopulations have been identified by their specific gene expression profiles, although virtually all definitive endoderm arises from a single source as revealed by fate mapping studies [14]:the rostral primitive streak.

**Figure 2:**
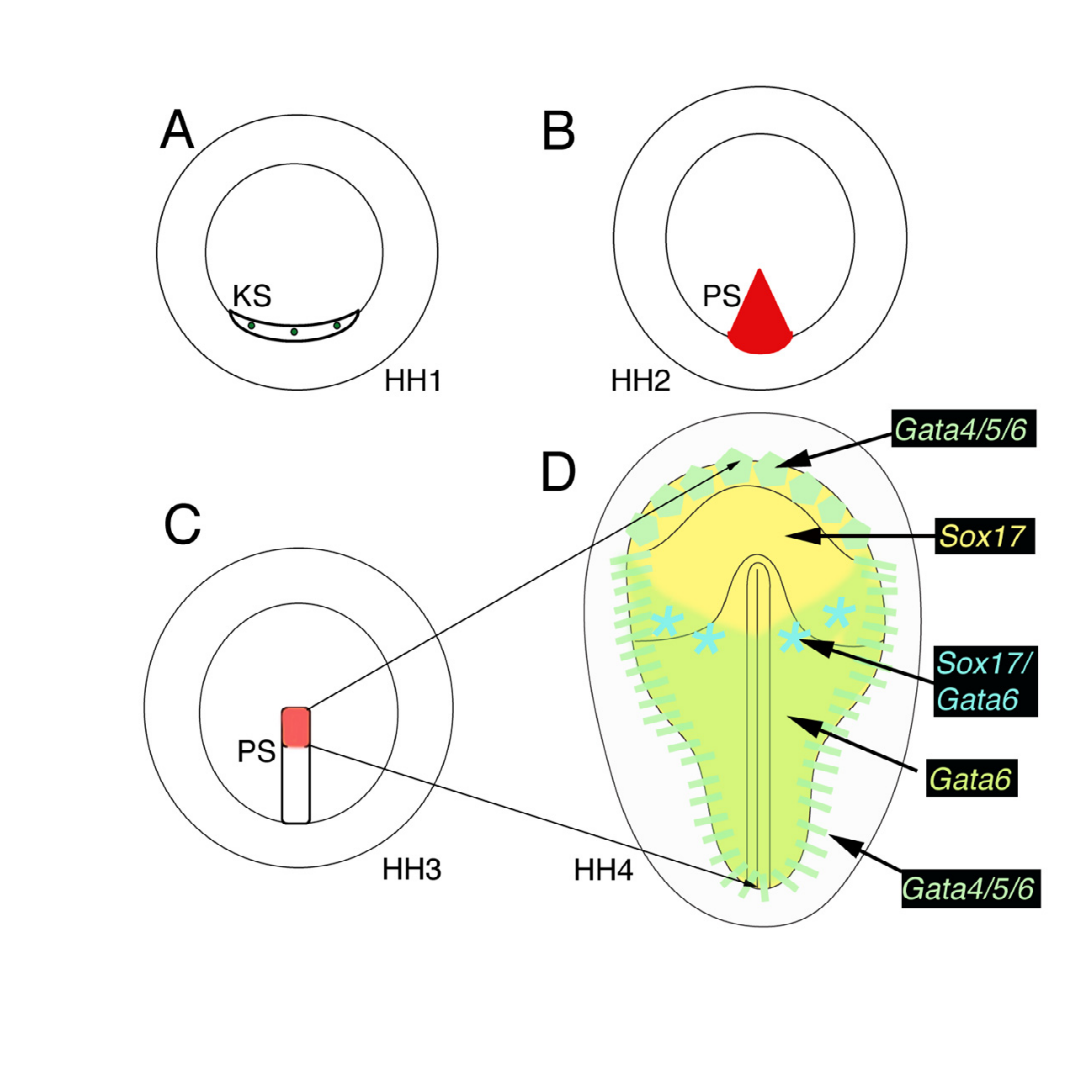
**Schematic drawing of definitive endoderm origin**. (A) Precursors of the primitive streak have been mapped to Koller's sickle (KS) at prestreak stages (green dots), and (B) are within the primitive streak at stage 2 (red, PS). (C) The rostral third of the extending streak (pale red) gives rise to the definitive endoderm, which ingresses displacing the hypoblast laterally and eventually forming the whole lower layer of the embryo (arrows to D). (D) Gene expression is regionally restricted within the lower layer. The rostral germ cell crescent and displaced hypoblast (pale green hexagons) and junctional mesendoderm (pale green bars) are *Gata4/5/6 *positive. Rostral definitive endoderm is *Sox17 *positive (yellow), with more caudal definitive endoderm expressing only *Gata6 *transcripts by stage 4 (green in area pellucida). The area of overlap at the boundary between *Sox17 *and *Gata6 *is marked by asterisks (blue).

*Sox17*, *Gata5 *and *Gata6 *are expressed in Koller's sickle in prestreak blastoderms. We have schematically illustrated prestreak Koller's sickle (Figure 2A), which contains precursors of the primitive streak that forms at stage 2 (Figure 2B). As the streak extends rostrally from stage 3 (Figure 2C), fate mapping identifies a rostral population that will give rise to the definitive endoderm. By stage 4, *Sox17*, *Gata5 *and *Gata6 *have distinct regional endoderm expression (Figure 2D), with *Gata5 *and *Gata6 *overlapping in a rostral crescent. *Sox17 *expression is restricted to rostral definitive endoderm, with an area of overlap at the boundary between *Sox17 *and *Gata6 *(data not shown) lateral to Hensen's node, whereas the caudal endoderm expresses only *Gata6*. These distinct combinations of expression may be important early markers/determinants of anterior to posterior cell types of the future gut tube. Fate mapping and in situ hybridization analysis will be required to formally test this possibility.

### Germ layer fate and molecular identity

Because the endoderm plays a critical role in patterning the developing embryo, it is important to establish when definitive endoderm first becomes committed to its germ layer fate. Kimura and co-workers [26] reported that "tip" cells (i.e., most rostral cluster of primitive streak cells) when transplanted more caudally in the primitive streak enter the lateral plate mesoderm and express a lateral plate marker, and more caudal streak cells when transplanted in place of "tip" cells contribute to the floor of the foregut and express *Sox2*. This result led these authors to conclude that germ layer identity was not fixed at the time prospective endoderm and mesoderm cells ingress through the primitive streak. Our identification and cloning of the chick definitive endoderm marker *Sox17 *enabled us to re-evaluate their studies and to extend them by examining other populations of primitive streak cells. This is important because 1) the primitive streak consists of multiple subpopulations, depending on its stage and rostrocaudal level, and the fates of different populations might become fixed at different times in development; and 2) "tip" cells are an ambiguous population to use in addressing the question of when endoderm and mesoderm germ layer fate becomes fixed. Kirby and co-workers [6] showed that cells in the most rostral part of the primitive streak (i.e., "tip" cells) contribute to the midline floor of the foregut (a finding confirmed by Kimura and co-workers [26]; see their Figure 7c) and subsequently (over the next 24 h) these cells leave the foregut floor to contribute to the endocardium and myocardium of the heart tube. Thus, these cells are unique and differ from all other cells in the rostrocaudal extent of the primitive streak in that they initially act like endoderm cells (entering the foregut) but later give rise to classical mesoderm tissues (the two early layers of the heart tube). Moreover, our expression studies (discussed above) show that "tip" cells are *Sox17*-negative (see our Figure 1D, arrow). To avoid the ambiguity presented by the use of "tip" cells, we choose a different prospective endoderm population, to analyze in our initial transplantation/grafting studies: those located just caudal to the tip cells but still within the rostral part of the primitive streak; these are known to form foregut endoderm almost exclusively [14]. Moreover, we used the quail-chick chimera system in our studies to address the degree to which streak cells are committed to their respective fates. This offers the distinct advantage that all donor (quail) and host (chick) cells can be unequivocally identified in chimeras after staining with a quail-specific nuclear antibody (QCPN), and no possibility exists for 1) failure to label all donor cells in tissue grafts, 2) the loss of label from some donor cells with further incubation (in which case they would be scored as host cells), or 3) the transfer of label from some donor to some host cells (in which case the latter cells would be scored as donor cells). Such complete and consistent fidelity of cell labeling has not been our experience with lipophilic dye labeling.

### Germ layer fate is fixed, but marker gene expression is labile

**Figure 3:**
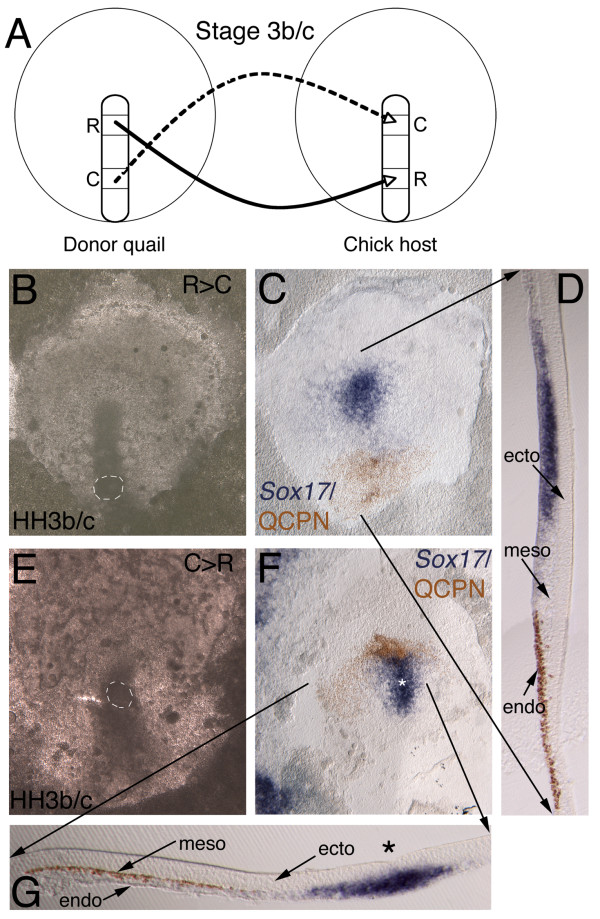
**Streak to streak quail-chick chimera transplants**. (B, C, E, F) Whole mount ISH, anterior to the top (*Sox17*, blue) and immunochemistry with anti-QCPN antibody (brown). (D, G) 50 μm gelatin sections. (A) Schematic depicting the experimental manipulation. Donor quail stage 3b/c with either rostral to caudal streak transplant (C-D) into chick host at same stage or caudal to rostral transplant (E-G). A second population of rostral streak cells, called "tip" cells (not diagrammed; see text and Figure 4) was also transplanted more caudally, and, conversely, caudal streak cells were transplanted to the tip site. (B) Host embryo at stage 3b/c within 1 hour of transplanting quail tissue from rostral to caudal streak, with fully integrated site highlighted by white dashed circle. (C) Same embryo after 4–6 hours incubation fixed and stained with *Sox17 *and anti-QCPN. (C, D) *Sox17 *is expressed in streak and more lateral migrating endoderm, but down regulated in transplanted cells, which are migrating normally. Sagittal section shows transplanted cells still retain endoderm germ layer position. (E) Caudal transplant integrated (white dashed circle) within rostral streak of stage 3b/c embryo and (F) after 6–4 hours incubation. Cells migrate normally away from streak. (G) Transverse section shows *Sox17 *in streak (asterisk), but absent from transplanted quail cells that nonetheless maintain mesoderm germ layer position.

To determine when the germ layer fate of prospective endoderm and mesoderm streak cells becomes committed we began by transplanting two groups of streak cells isochronally and heterotopically (i.e., stages 3b/c: ~8 hours incubation; rostral streak to caudal streak and vice versa, with the former population excluding the "tip" cells; Figure 3A). Based on our expression studies (discussed above) and fate mapping studies [27,28,14-17], the rostral streak cells were expected to be *Sox17*-expressing prospective endoderm cells (Figure 1C, D), and the caudal streak cells were expected to be *Sox17*-negative/*Wnt8c*-positive prospective mesoderm cells (Figure 1C, D and 6 inset).

After transplantation of quail rostral primitive streak into a more caudal streak position, chimeras were incubated for 4–6 hours (n = 9) (Figure 3A, B–D). *Sox17 *expression would be expected to be detected in the transplanted cells for 12 hours following incubation, as down regulation of *Sox17 *in definitive endoderm does not occur until 12 hours after stage 3c when the tissue reaches stage 5 (~20 hours incubation), as described in our expression studies above. The transplanted tissue integrated into the caudal streak within an hour (Figure 3B), and transplanted cells migrated away from the streak into the area pellucida during the re-incubation period (Figure 3C). *Sox17 *expression was not maintained in the grafted tissue, although it was strongly expressed in the anterior streak and definitive endoderm of the chick host (Figure 3C, D). However, the quail-positive prospective endoderm cells migrated into their normal position as the ventral-most epithelial cell layer (Figure 3D). Caudal, non-*Sox17 *expressing streak cells transplanted to more rostral streak (n = 8) (Figure 3A, E–G), did not turn on *Sox17 *in their new position (Figure 3F, G), although they too were able to migrate away from the streak (Figure 3F). The prospective mesoderm cells migrated into their normal mesoderm germ layer position, forming a mesenchymal layer between the ectoderm and endoderm (Figure 3G).

Thus, altering the rostral and caudal position of cells within the streak by transplanting tissue is unable to induce *Sox17 *in caudal non-*Sox17 *expressing prospective mesoderm cells, nor is *Sox17 *expression maintained in definitive endoderm cells outside of their normal environment, but cells establish a normal germ layer morphology and position in both cases that is commensurate with their original level of origin in the streak. We conclude that prospective endoderm and mesoderm cells in the streak at stages 3b/c are already committed to their germ layer fate, but lose their molecular identity when transplanted heterotopically.

**Figure 4:**
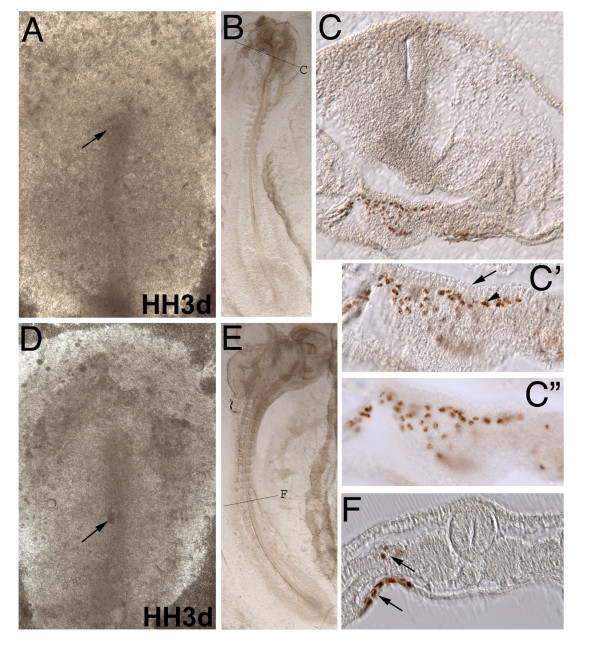
**Quail-chick chimera tip cell streak transplants**. (A-C) Caudal quail streak cells transplanted to the chick rostral tip of the streak at stage 3d (A-0h, arrowed) and incubated for 22 hours in EC culture, labeled with QCPN antibody to identify donor quail cells (B). Quail cells in rostral head of whole mount embryo at stage 10. Line indicates level of transverse section (C), 50 μm gelatin section through head with QCPN positive cells maintaining their mesoderm fate. (C' and C") High magnification (10x) images of quail cells from C, with and without Hoffman modulation contrast optics, respectively. Transplanted cells maintain their mesodermal fate (arrowhead) and are mostly excluded from the foregut and endoderm (arrowed). (D-F) Quail rostral streak "tip" cells transplanted into chick mid-streak (D, arrowed, 0 h). (E) Cells migrate laterally after 22 hours of incubation (stage 12 whole mount embryo). Black line indicates level of transverse section F. The donor quail cells maintain their endodermal fate (arrowed), with a few mesoderm cells found in the intermediate mesoderm (arrow).

These results differ from those of Kimura and co-workers in that in our experiments we demonstrate that endoderm and mesoderm germ layer identity is established by the early primitive streak stage, whereas in their experiments germ layer identity seems to be labile at this time. However, it is possible that because the populations of cells transplanted differed in the two studies, differing populations might have different properties. Hence, we repeated their experiments using identical sized grafts and the exact same populations of cells using the chick-quail chimera system. Seven chimeras were constructed: 3 in which more caudal streak cells were grafted into the tip region, and the converse experiment, with 4 chimeras in which "tip" cells were grafted more caudally. In 3 out of 3 cases in which more caudal cells were grafted to the "tip" region, all cells contributed to the ventral-most mesoderm (Figure 4A–C, C', C"), consistent with their original level of origin in the streak (i.e., a level that gives rise to prospective mesoderm). In 3 out of 4 cases in which "tip" cells were grafted more caudally, all cells populated the gut endoderm, consistent with their original level of origin in the streak (i.e., a level that largely gives rise to prospective endoderm), but in one case (the one illustrated in Figure 4D–F) although most cells populated the gut endoderm, a few cells also populated the overlying intermediate mesoderm. As the rostral-most primitive streak also contains mesoderm cells (prospective head and heart mesoderm) as well as endoderm cells (Figure 4B and Lawson et al., in preparation), and mesodermal cells readily change their subdivision fate when heterotopically or heterochronically transplanted [29,30], it is not unlikely that some prospective mesoderm cells where included in the "tip" cell population transplanted in this one embryo. Thus, all of our experiments, including those that repeat the study of Kimura and co-workers [26] lead to the conclusion that endoderm and mesoderm germ layer fate is committed in the primitive streak at the time of gastrulation in the chick. Furthermore, our data supports the widely accepted view that endoderm is the first layer to become committed and does so during early stages of gastrulation [31,32].

It is unknown why our results on the commitment of germ layer fate differ from those of Kimura and co-workers [26]. Although we attempted to precisely replicate their experiments using "tip" cells, possible sources of variation in the two studies that might account for the differences could include slight differences in stages of embryos used, the exact populations of cells transplanted, the exact position at which cells were placed in the primitive streak, and potential artifacts associated with dye labeling or intraspecies grafting. Nevertheless, it is important to point out that because the tip of the streak contains cells fated to enter the heart [6] (a classic mesodermally derived tissue), they could actually represent prospective mesoderm cells, not prospective endoderm cells, so heterotopically transplanting these cells might not assess whether a germ layer fate change has really occurred. Similarly, substitution of "tip" cells with more caudal prospective mesoderm cells also might not assess whether a germ layer fate change occurred because cells entering the midline floor of the foregut are known to only temporarily reside there before leaving the gut tube and forming the inner and outer layers of the heart tube [6]. Hence, our experimental design for providing a definitive test of the fate of prospective endoderm and mesoderm cells of the streak included transplanting the more caudal rostral streak cells, which contains prospective endoderm cells *not *contributing to the midline floor of the foregut [6,14]. This experiment and the converse experiment, show unequivocally that the fate of these two populations of cells is committed at early primitive streak stages.

In contrast to our results on germ layer fate, which contradict those of Kimura and co-workers [26], our results on marker gene expression complement and extend those of these investigators, who showed that marker gene expression patterns are labile. Similarly, we show using another marker and testing another less-problematic prospective endoderm cell population (i.e., prospective endoderm cells caudal to the "tip" cells) that 1) *Sox17*-positive rostral cells when placed more caudally, loose *Sox17*-expression but still contribute to the endoderm layer (i.e., their normal fate); and 2) *Sox17*-negative caudal cells when placed more cranially fail to express *Sox17 *but still contribute to the mesoderm layer (i.e., their normal fate).

### Inductive and repressive blastoderm signals regulate endoderm and mesoderm gene expression

**Figure 5:**
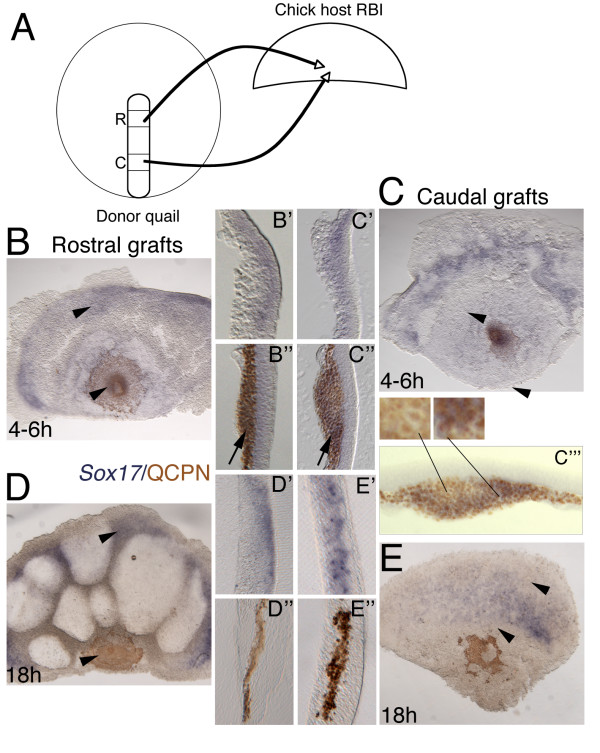
**Streak to RBI quail-chick chimera transplants and *Sox17 *expression**. ISH (*Sox17*, blue), and immunochemistry with anti-QCPN antibody (brown). Anterior to the top, (D, F) 50 μm gelatin sections, with level indicated by arrowheads. (A) Schematic of experiment. Donor quail tissue from rostral or caudal streak transplanted to rostral blastoderm isolate (RBI). (B) Rostral quail streak explanted into RBI for 4–6 hours has *Sox17 *expression in host RBI (B') and most of the donor QCPN positive cells (B"). (C) Similarly, when caudal streak cells are explanted into the RBI, the RBI has endogenous expression of *Sox17 *in area opaca (C') and most of the QCPN positive transplanted cells express *Sox17 *within 4–6 hours (C", C"'). C"' is the same section as C" taken without Hoffman contrast optics with lines to 10× magnification inset images to aid visualization. The central grafted quail cells are QCPN positive/*Sox17 *negative (inset left image has QCPN cell nuclei surrounded by *Sox17 *negative cytoplasm; also shown by whole mount images of *Sox17 *expression at stages 3c-4+ in which the node is clearly unlabeled; Figure 1) and the outer quail cells are QCPN and *Sox17 *positive (right inset image has QCPN positive nuclei surrounded by *Sox17 *positive blue cytoplasm). Note cells double labeled for *Sox17 *and QCPN (arrow) in B" and C". (D) Following overnight incubation only the endogenous RBI expression remains (D'), with the quail explant now negative for *Sox17 *expression (D"). (E) Overnight incubation of a caudal explant reveals that *Sox17 *is expressed only in endogenous chick cells (E'), whereas the QCPN positive cells have downregulated *Sox17 *expression (E").

During gastrulation, definitive endoderm exits the anterior one-third of the primitive streak from stage 2 when the streak forms, through to the extended streak stage (stage 4). Definitive endoderm spreads rostrally and laterally, displacing hypoblast cells to the interface between the area pellucida and area opaca where the hypoblast is fated to form extraembryonic tissue [1]. Because the rostral streak tissue was not able to maintain *Sox17 *expression in a more caudal position, we questioned whether the rostral blastoderm maintained the molecular identity of definitive endoderm as this tissue migrated from the streak. To test this we excised rostral *Sox17*-positive and caudal *Sox17*-negative streak cells from quail at stages 2 to 3d (4–10 hours incubation), and transplanted the tissue to stage 1 or 2 chick host rostral blastoderm isolates (RBIs) (4 hours incubation) (Figure 5A). The explants were incubated for either an additional 4–6 hours or overnight (~18 hours) and analyzed for *Sox17 *expression to determine a) whether *Sox17 *expressing anterior streak definitive endoderm is maintained, and b) the RBI is able to induce *Sox17 *markers in more caudal mesodermal tissue. After 4–6 hours of incubation rostral explants maintained *Sox17 *expression (20/21, Figure 5B, B', B"), but down regulated expression after overnight incubation (2/12, Figure 5D, D', D"). Cells migrated away from the graft site as expected. This raises the question of whether the RBI is permissive or instructive in regard to *Sox17 *expression, i.e. are endoderm cells already committed to express *Sox17 *when they exit the streak, or is a signal from the RBI required for the cells to express *Sox17*.

To answer this question specifically, *caudal *quail donor streak tissue was excised and transplanted to chick RBIs (Figure 5C). This tissue is negative for Sox17 expression as determined by taking 5 donor embryos from which rostral and caudal grafts were remove and subjecting the donor embryo to in situ hybridization for *Sox17 *(data not shown). In whole mounts in all 5 cases, it was evident that rostral grafts were taken from an area expressing *Sox17 *but caudal grafts were taken from an area that was caudal to the most caudal expressing level. After transplantation, cells from the more caudal transplanted streak turned on *Sox17 *expression in all cases (n = 7, Figure 5C, C', C", C"', and 5C"' insets), but were unable to maintain expression after overnight incubation (0/7, Figure 5E, E', E"). This experiment suggests that the RBI is able to induce the expression of *Sox17 *in tissue fated to be mesodermal, but is not able to maintain the expression over time. This is in line with the normal expression of *Sox17*, where definitive endoderm expresses *Sox17 *for 8–12 hours after leaving the streak, but then down regulates expression to a small subset of cells in the prechordal plate endoderm and a crescent of cells expressing *Sox17 *in the liver primordium (Figure 1A–G). Together these data indicate that the RBI is *instructive *for *Sox17 *expression in cells derived from both rostral and caudal streak, but this ability is not maintained after stage 5. The responsible instructive signals could potentially reside in either the rostral ectoderm, hypoblast or area opaca. Given the patterning role of the midline axial derivatives of Hensen's node, prechordal plate endoderm and anterior notochord [1,33], it is plausible that instructive signals from rostral tissue, required for inducing/maintaining *Sox17 *expression, are turned off at stage 5, as the prechordal plate endoderm and anterior notochord extend rostrally from the streak into this region.

**Figure 6:**
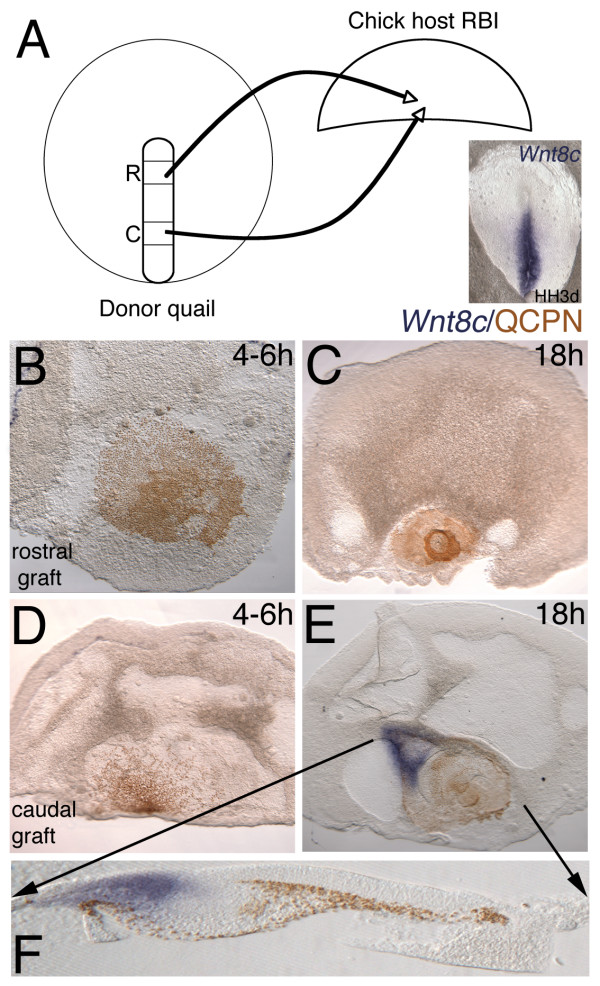
**Streak to RBI quail-chick chimera transplants and *Wnt8c *expression**. ISH (*Wnt8c*, blue) and immunochemistry (anti-QCPN antibody, brown). Anterior to the top, (F) Level of 50 μm gelatin section marked by black arrows. (A) Schematic of experiment with donor quail tissue from rostral or caudal streak transplanted to chick rostral blastoderm isolate (RBI) and analyzed using *Wnt8c *ISH marker. Whole mount inset shows *Wnt8c *is restricted to the caudal two-thirds of the primitive streak and ingressed mesoderm directly adjacent to the primitive streak. (B, C) As expected, *Wnt8c *is not expressed in transplanted rostral streak or RBI at either 4–6 hours or overnight incubation. (D) *Wnt8c *expression is down regulated in caudal cells transplanted to RBIs after 4–6 hours. (E) Caudal streak transplanted to the RBI is unable to express *Wnt8c *after overnight incubation, but the explant induces *Wnt8c *in the chick host tissue (F) Section with *Wnt8c *expression in chick tissue and negative for expression in quail cells.

### Rostral Blastoderm Isolates (RBIs) initially represses the mesoderm marker Wnt8c

**Figure 7:**
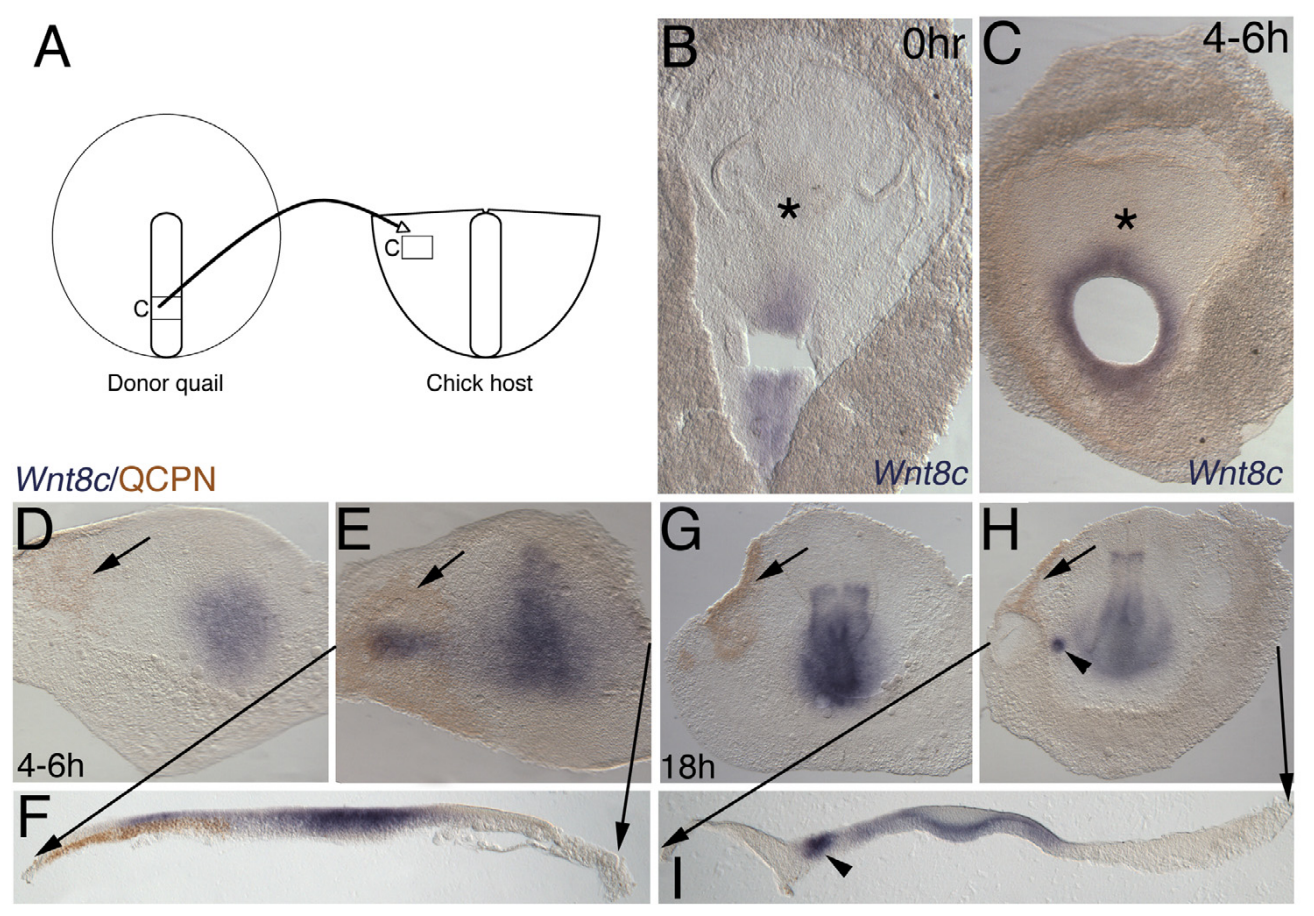
**Streak to CBI quail-chick chimera transplants**. (B-I) ISH (*Wnt8c*, blue) and (D-I) immunochemistry (anti-QCPN antibody, brown). Anterior to top. (F, I) Black lines from E and H mark level of 50 μm transverse gelatin sections. (A) Schematic of experiment showing caudal streak isolate transplanted lateral to the streak in the area pellucida. (B) Whole mount *Wnt8c *ISH of quail donor following removal of the explant, showing that donor tissue is *Wnt8c *positive. (C) Whole mount donor quail after 4–6 hours of incubation showing that area around explant remains *Wnt8c *positive. (D-I) In no case does explanted tissue express *Wnt8c *after 4–6 hours or overnight incubation. (D-F) 4–6 hours of incubation, quail graft integrated and spreading (arrowed). (D) Explanted quail streak cells with no ectopic *Wnt8c *(E, F) Ectopic *Wnt8c *induced in overlying ectoderm reminiscent of an ectopic streak transverse to normal orientation. (G-I) Caudal streak explant into lateral area pellucida of caudal blastoderm isolate. Overnight incubation with quail cells now restricted to edge of CBI (arrowed). (G-I) Following overnight incubation no quail cells express *Wnt8c*. (H, I) In only in 2 cases is a small spot of ectopic *Wnt8c *still detectable in ectoderm (arrowhead).

*Wnt8c *marks the streak and ingressing mesoderm, but is excluded from the anterior-most portion of the streak from which definitive endoderm ingresses [34,1]. We asked whether the rostral blastoderm, which promotes *Sox17 *identity in rostral definitive endoderm, plays a role in repressing more caudal identity in cells exiting the streak. The experiment was performed as described for *Sox17 *and analyzed for *Wnt8c *expression (Figure 6A). Rostral streak does not normally express *Wnt8c *and in no case was *Wnt8c *induced in the quail cells grafted into the rostral blastoderm isolate (Figure 6B: 4–6 hours, 0/5; Figure 6C: overnight, 0/5). Caudal mesodermal cells express *Wnt8c *at the time of excision (Figure 6A inset, and Figure 7B), but quail cells transplanted to the RBI down regulate *Wnt8c *(n = 6) after 4–6 hours of incubation (Figure 6D). These data indicate that the RBI is either unable to maintain, or actively inhibits, *Wnt8c *expression. Interestingly, after overnight incubation, 8/8 RBIs express *Wnt8c *in the host tissue, but not in quail donor tissue (Figure 6E, F). The most likely explanation for this phenomenon is that a change occurs in signaling capability of stage 5 chick embryos, so that the RBI is no longer able to repress Wnt8c, thereby freeing the transplanted streak cells to act like an organizer and induce ectopic *Wnt8c*. However, this does not explain why the transplanted quail cells themselves are unable to express *Wnt8c*.

### Early Caudal Blastoderm Isolates (CBIs) is permissive for *Wnt8c *expression

To further test the idea that rostral and caudal embryo are molecularly different we transplanted caudal streak to the lateral area pellucida of CBIs at stages 3b/c (Figure 7A). Whole mount quail donor embryos expressed *Wnt8c *at 0 hours (Figure 7B), 4–6 hours of incubation (Figure 7C) and overnight incubation (not shown) in the streak around the area of excision. At 4–6 hours of incubation chick host ingressing mesoderm expressed *Wnt8c *normally, with 5/9 CBIs having no ectopic *Wnt8c *expression (Figure 7D) and 4/9 CBIs having broad ectopic *Wnt8c *expression in the host chick ectoderm tissue overlying the graft, but in all cases transplanted quail cells were negative for *Wnt8c *(Figure 7E–F). The ectopic expression is reminiscent of a transplanted ectopic streak oriented transversely to the endogenous streak. Following overnight incubation, most embryos had no ectopic *Wnt8c *(Figure 7G), with only 2/14 embryos having small ectopic *Wnt8c *expression spots remaining in the chick ectoderm (Figure 7H, I). We interpret this as repression of ectopically induced streak and in all case the CBIs had no *Wnt8c *expression in the grafted quail cells (Figure 7H, I). The CBI is a permissive environment for *Wnt8c *expression in the streak and ingressing mesoderm, but transplanted cells lose their *Wnt8c *expression, just as in RBIs. However, ectopic *Wnt8c *expression is induced in the ectoderm overlying the graft. The CBI and RBI differ in their effect on transplanted caudal streak in that the RBI chick tissue expresses *Wnt8c *after overnight incubation, whereas caudal tissue expresses ectopic *Wnt8c *in about half of cases at 4–6 hours, with this ability progressively restricted following overnight incubation. This result indicates that the CBI changes over time and becomes less permissive of *Wnt8c *expression outside its normal domain. Whereas the RBI, which lacks ingressing axial mesoderm, does not have a mechanism for repressing signals that induce ectopic *Wnt8c *after overnight incubation.

## Conclusion

Our results demonstrate that germ layer identity is fixed at the time populations of endoderm and mesoderm cells ingress through the primitive streak, whereas their gene expression patterns remain labile. In addition, our results show that inductive and repressive signals are present, and that these signals regulate gene expression of both ingressed endoderm and mesoderm cells. Thus, gastrula cells display elements of both pre-patterning and plasticity, with endoderm the first germ layer becoming committed to its fate during early gastrulation stages.

## Methods

Incubation, harvesting, staging, in situ hybridization (ISH) and vibratome gelatin sectioning were performed according to our standard protocols as described previously [35].

Quail eggs (Strickland Farms) and chick eggs (Utah State University) were incubated to required stages. Quail and chick blastoderms were removed from the vitelline membranes under saline. To prevent tissue damage by prolonged exposure to saline, the blastoderms were submerged in Leibowitz L15 medium (Gibco). Some chick blastoderms were cultured intact (after removal of most of the area opaca) on a substrate of the semi-solid agar/albumen medium in 35 mm plastic petri dishes [36]. Other chick blastoderms were transected into rostral and caudal pieces prior to culture. Quail blastoderms were used as donor tissues for transplantation studies. In one set of experiments (see Figure 3 and 4), donor quail tissue was excised from the primitive streak and transplanted into whole chick blastoderms (host tissue) that had been placed ventral side up on agar/albumen dishes. Donor tissue was transplanted isochronically but heterotopically (i.e., caudal donor streak to two levels of rostral host streak, and two levels of rostral donor streak to caudal host streak; only one level of the rostral streak transplants is shown in Figure 3–the other level consisted of the "tip" cells of the streak seen in Figure 4). In a second set of experiments (see Figure 5, 6, 7), donor quail tissue was excised from the primitive streak and grafted into transected chick blastoderms (host tissue rostral or caudal blastoderm isolates) between the epiblast and hypoblast. In all experiments, as much of the media as possible was removed to ensure good contact between the tissue and agar/albumen substrate. Several small petri dishes were placed into a larger petri dish with a moistened filter paper base to prevent desiccation of the tissue. Embryos were further incubated in humidified incubators with 5% CO_2 _for 4 hours to overnight at 38°C. After fixation, embryos were analyzed by ISH and then immunocytochemistry using the quail specific antigen QCPN. The QCPN hybridoma developed by Bruce M. and Jean A. Carlson was obtained from the Developmental Studies Hybridoma Bank developed under the auspices of the NICHD and maintained by The University of Iowa, Department of Biological Sciences, Iowa City, IA 52242.

## Authors' contributions

SCC performed the quail-chick chimera studies, oversaw the project, prepared the images and drafted the manuscript. KM performed the ISH analysis, prepared ISH images and assisted in drafting of the manuscript. QC performed the molecular genetic analysis, cloning and preparation of molecular reagents used in the ISH analysis. GCS conceived the study, participated in its design and helped draft the manuscript. All authors read and approved the final manuscript. All authors participated in data analysis and interpretation.

## Supplementary Material

Additional file 1 Whole mount *Sox17 *expression stages 5–24. Composite image of Sox17 in situ hybridization from stages 5–24. Click here for file
