# Supplementary material for: Specification of germ layer identity in the chick gastrula
Source: BMC Dev Biol. 2007 Jul 30;7:91. doi: 10.1186/1471-213X-7-91 (PMC2000891; doi:10.1186/1471-213X-7-91)
Supplement: Additional file 1 — Whole mount Sox17 expression stages 5–24. Composite image of Sox17 in situ hybridization from stages 5–24. [file 1471-213X-7-91-S1.pdf]

Extended stage analysis of *Sox17* gene expression: Whole mount *Sox17* expression stages 5-24

Because of its specific expression pattern in the early embryo and its importance in endoderm formation in other species, and to determine to what extent *Sox17* serves as an endoderm marker we have extended our analysis of *Sox17* to include stages 5-24.

Whole mount (A, B, D, F, I) in situ hybridization (ISH) and 50 μm gelatin sections (C, E, G, H, J-L). Anterior to the top. Lines on whole mount images show level of accompanying section. (A, C) Stage 5, following ingression and extension of the notochord, *Sox17* expression is down regulated to a subset of prechordal plate endoderm (pcpe) cells (arrow) and a crescent of endoderm at the level of the future head fold. Arrowhead marks pcpe cells in sagittal section (C). This expression is reminiscent of *Hex* at stage 5 and thus we performed double ISH with *Hex* and *Sox17* to determine their relationship (B, D, E). (B) Double ISH with *Sox17* (blue, arrow) as a subset of cells within the *Hex* (red) domain. The subset of cells within the pcpe expresses *Sox17* within the broader *Hex* expressing domain, whereas the crescent of cells in the head fold are adjacent to *Hex*, with *Sox17* being more rostral and extend less laterally than *Hex* (B, D). (D) Double ISH showing *Sox17* (blue) adjacent to *Hex* (red) in head fold at stage7/8 marking the putative liver domain (pcpe arrowed). (E) Section shows both markers in endoderm layer. Arrowhead marks pcpe within *Hex* domain of the head fold. (F) Stages 10-17, with embryo shown stage 14, ventral view. Detectable transcripts are in the presumptive liver rudiment of a whole mount embryo at the level of the anterior intestinal portal (AIP, arrow), which marks the transition between the fore- and mid-gut levels. (G) Sagittal section from F and (H) transverse section from similar embryo with transcripts restricted to two patches of endoderm (endo). Expression is restricted to the endoderm layer of the anterior intestinal portal as the medial edges come together to form the caudal liver rudiment. Dorsal foregut (df, arrow) has weak *Sox17* staining. (I) Stages 18-24 (embryo depicted, stage 21) expression is detected in the head and two restricted domains. In whole mount, a line of expression extends from the anterior notochord at the level of the midbrain, caudally to the spinal cord (arrowheads). (J) Transverse section, telencephalon to the left, transcripts in medial peri-ocular mesenchyme (mpom) of diencephalon (arrow). (K) Hindbrain level, neural tube to the right. Bilateral staining in 5th branchial pouch (arrow) marking ultimobranchial bodies (ubb) that give rise to 'C' cells (calcitonin producing cells). Arrowhead marks mesenchyme cells at base of floorplate between neural tube and notochord. Transcripts are detected in the mesenchyme between the ventral neural tube and notochord, which is itself negative for transcripts. (L) Transverse section at level of forelimb, spinal cord to the right, unilateral group of cells (arrowed) marking caudal liver rudiment (clr). Taken together, these data suggest that *Sox17* is only a transient marker of ingressing definitive endoderm during gastrulation. Several restricted sites of expression in the developing embryo suggest that restricted expression of *Sox17* may be required for development of various tissues, including the liver.

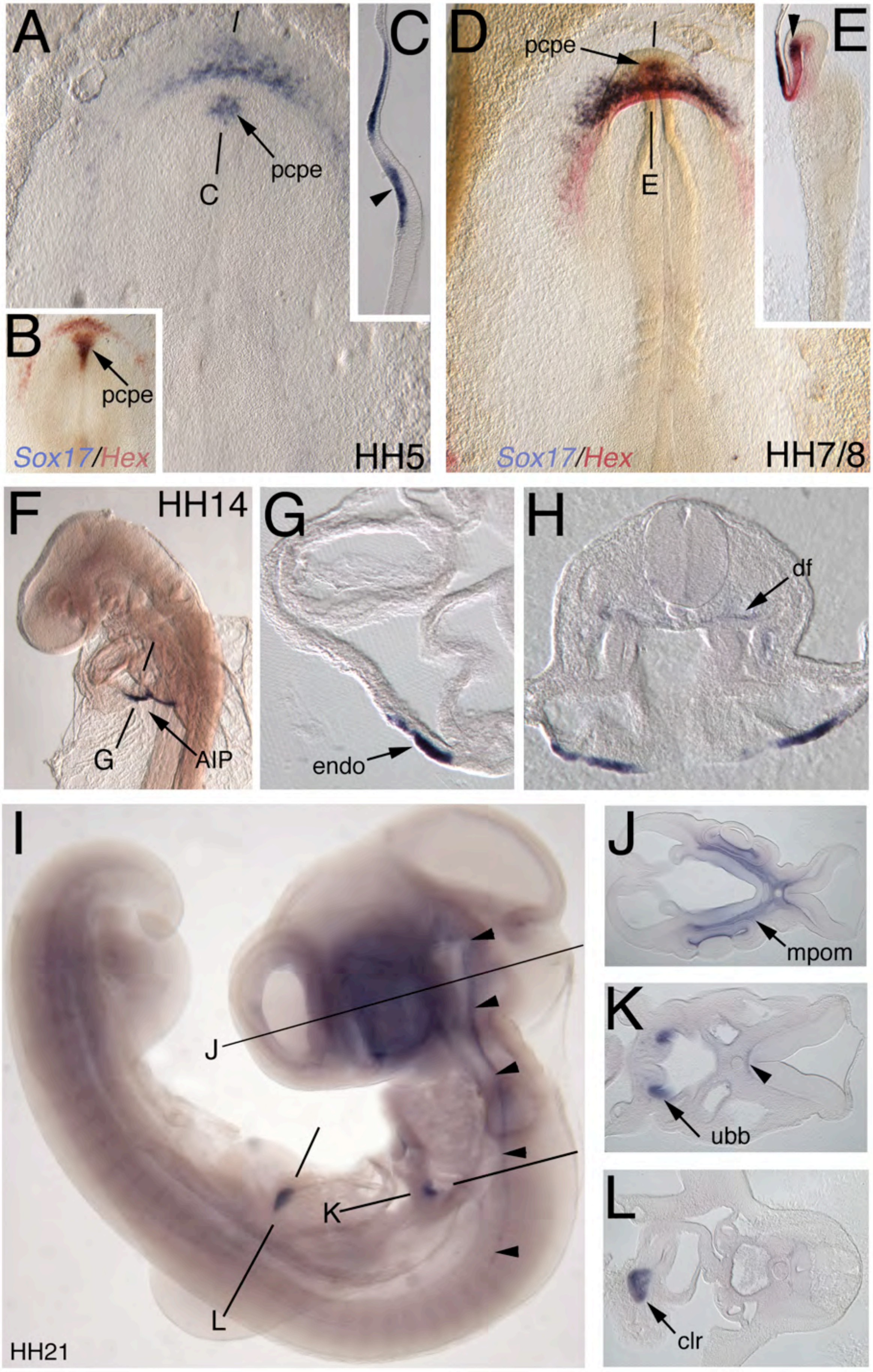
A
C
pcpe
C
B
pcpe
Sox17/Hex
HH5
D
pcpe
E
E
Sox17/Hex
HH7/8
F
HH14
G
AIP
G
endo
H
df
I
J
K
L
HH21
J
mpom
K
ubb
L
clr
